# Enhancement of extinction memory by pharmacological and behavioral interventions targeted to its reactivation

**DOI:** 10.1038/s41598-017-11261-6

**Published:** 2017-09-08

**Authors:** Josué Haubrich, Adriano Machado, Flávia Zacouteguy Boos, Ana P. Crestani, Rodrigo O. Sierra, Lucas de Oliveira Alvares, Jorge A. Quillfeldt

**Affiliations:** Psychobiology and Neurocomputation lab and Neurobiology of Memory lab. Neurosciences Graduate Program, Universidade Federal do Rio Grande do Sul, Porto Alegre, Brazil

## Abstract

Extinction is a process that involves new learning that inhibits the expression of previously acquired memories. Although temporarily effective, extinction does not erase an original fear association. Since the extinction trace tends to fade over time, the original memory can resurge. On the other hand, strengthening effects have been described in several reconsolidation studies using different behavioral and pharmacological manipulations. In order to know whether an extinction memory can be strengthened by reactivation-based interventions in the contextual fear conditioning task, we began by replicating the classic phenomenon of spontaneous recovery to show that brief reexposure sessions can prevent the decay of the extinction trace over time in a long-lasting way. This fear attenuation was shown to depend both on L-type calcium channels and protein synthesis, which suggests a reconsolidation process behind the reactivation-induced strengthening effect. The extinction trace was also susceptible to enhancement by a post-reactivation infusion of a memory-enhancing drug (NaB), which was also able to prevent rapid fear reacquisition (savings). These findings point to new reactivation-based approaches able to strengthen an extinction memory to promote its persistence. The constructive interactions between extinction and reconsolidation may represent a promising novel approach in the realm of fear-related disorder treatments.

## Introduction

Memory retrieval is a dynamic phenomenon that can, given the right conditions, trigger two distinct processes, reconsolidation or extinction. Following a brief retrieval session in the same training context, a previously consolidated memory may enter a labile state that requires *de novo* protein synthesis in order to re-stabilize and persist, a process called reconsolidation^1^. However, prolonged, non-reinforced retrieval sessions can induce extinction^2^. Although the reconsolidation of fear memories have been extensively studied^3, 4^, very few studies have so far investigated the possibility that an extinction trace would undergo reconsolidation following retrieval^5–7^, and its possible outcomes and clinical applications are currently unexplored^8^.

Extinction decreases conditioned responses through a process that entails the consolidation of a new inhibitory memory; it is thought not to be unlearning or erasure of the original trace^9, 10^. Extinction-based therapies are commonly employed to hinder aversive responses in patients with fear-related disorders such as posttraumatic stress disorder^11, 12^. Despite being an effective intervention, the relapse of fear symptoms is often reported^13^ showing that, compared to robust fear memories, extinction is less enduring and prone to decay. Fear responses can easily recover due to several relapse processes, such as spontaneous recovery, reinstatement, renewal, and rapid reacquisition^14, 15^. Accordingly, it is of great importance to find better approaches to enhance extinction strength and persistence.

Despite both reconsolidation and extinction being triggered by retrieval, they are distinct processes. Behaviorally, reconsolidation is usually engaged by brief re-exposures to the conditioned stimulus (CS) whereas extinction requires longer ones. In addition, the induction of reconsolidation is modulated by other contextual and cognitive factors collectively known as boundary conditions^16^. By varying the duration of exposure to the CS, an amnestic agent will selectively impair either the reconsolidation of the original trace, or the consolidation of the extinction trace^17–23^. The fact that both processes do not take place simultaneously suggests a trace dominance effect, in that the dominant trace will be the first, if not the only one, affected by any interference. Trace dominance also occurs when previously consolidated fear and extinction traces coexist, affecting their retrieval. In such conditions, re-exposure to the CS, which can potentially activate both fear and extinction traces, will result in the expression of the dominant memory and in the inhibition of the other one^2^. As discussed above, initially, extinction is dominant and easily suppresses fear. However, with time the original fear trace overcomes the inhibition by extinction and becomes dominantly expressed again^14^. In addition, there is a double dissociation between the two process regarding molecular markers such as Zif268^24^, calcineurin^19, 25^ and BDNF^26^, indicating that both processes do not occur in parallel.

It is often suggested that the functional role of the destabilization-restabilization process behind reconsolidation is to allow memory to update in order to retain its predictive and adaptive relevance^3, 27–29^. For instance, memory content can be updated through the incorporation of novel information^30–34^. In addition, several studies reported that reconsolidation may mediate memory enhancement and strengthening^30, 35–39^ as well memory attenuation (without extinction) of aversive experiences^34, 40^. Interestingly, there are studies showing that in some tasks reconsolidation only happens when memory is not yet at an asymptotic level^41–43^, further highlighting its role in the strengthening of the memory trace. However, so far it is unknown whether a consolidated extinction trace can be modified by reconsolidation.

Reconsolidation can open a window to allow the pharmacological modulation the reactivated memory. This would parallel classic experiments in which the post-reactivation infusion of amnestic agents impair memory^1^, or at least effectively diminish fear responses in phobias^44^. Also, post-reactivation interventions that promote reconsolidation can lead to enhanced performance^45–47^. Hence, specific compounds administered during the window of memory lability during reconsolidation allows for positive or negative modulation of memory strength.

Given its clinical relevance, there is considerable interest in the development of more efficient extinction-based approaches^48^. Extinction memories are effective in transiently suppressing fear responses but the fear returns easily^2^. Stemming from the fact that the retrieval-driven process of reconsolidation can lead to memory strengthening, either behaviorally^30, 35–39^, or pharmacologically^45–47^, we hypothesize that if an extinction memory is reactivated, it may undergo a reconsolidation process and be positively modulated by behavioral and pharmacological interventions, resulting in an increased resistance to relapse.

To verify this, we evaluated the effect of brief re-exposures to the conditioned context in animals that were previously trained in contextual fear conditioning (CFC) and submitted to extinction. We found that spontaneous recovery was observed 2 weeks after extinction, but periodical reactivation sessions were able to delay fear trace resurgence for at least 4 weeks. This effect was shown to depend upon L-type voltage-gated calcium channels (L-VGCC), and a reactivation one day after the extinction session was shown to cause the extinction trace to transiently become labile in a protein-synthesis dependent manner. All taken together, the data strongly suggests a reconsolidation process acting upon the extinction trace. Also, in a protocol employed to investigate fear rapid reacquisition - another post-extinction relapse process^49^ - a single post-reactivation infusion of sodium butyrate (NaB), a HDAC inhibitor that positively regulates neuronal plasticity^50^, was able to enhance the extinction memory to the point of resisting fear recovery. The evidence shows that the extinction trace can be effectively strengthened by reactivation-based interventions.

## Results

### Experiment 1: Extinction memory only transiently inhibits fear expression

**Figure 1:**
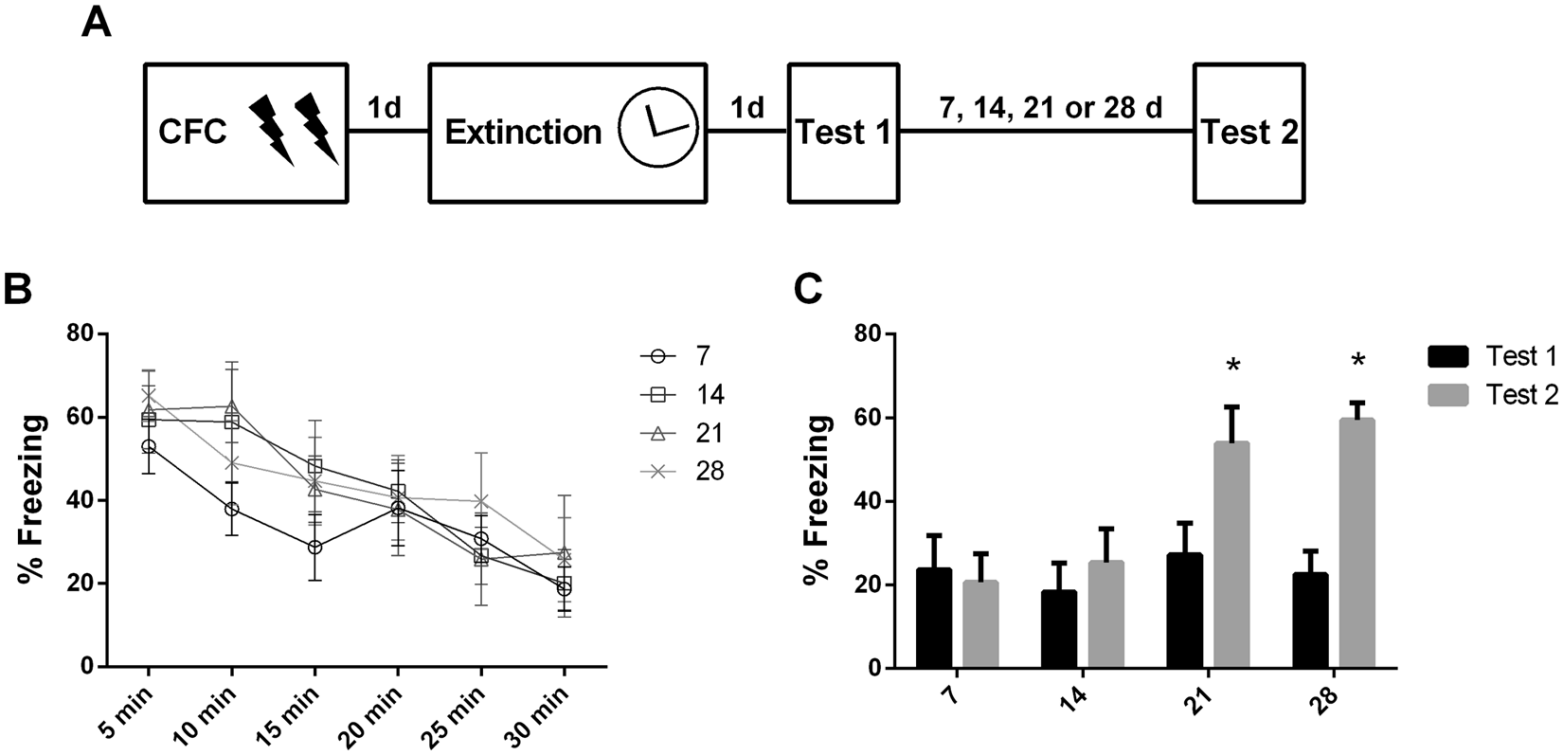
Extinction memory fails to suppress fear permanently. (**A**) Schematic representation of the experimental procedures. Fear conditioned rats were submitted to 30-min extinction session and were tested 24 hours later. A second test was conducted 7, 14, 21 or 28 days after test 1 (N = 6 /9 per group). (**B**) Freezing levels during extinction sessions. (**C**) Freezing levels during tests. (*) Significant difference between tests 1 and 2 (P < 0.05, Repeated-Measures ANOVA followed by Tukey’s *post-hoc* test).

Extinction is new learning that temporarily suppresses a previously acquired memory. Thus, following extinction, two opposing memories co-exist and compete for expression. Initially, the extinction memory is dominant over the fear trace and thus able to inhibit its expression. However, this suppression is not permanent. As time elapses, fear memory overcomes extinction inhibition and aversive behavioral responses return. This process is called spontaneous recovery^15^. First, we assessed the temporal profile of spontaneous recovery in our CFC protocol. Accordingly, animals were fear conditioned and 24 hours later underwent extinction training. The next day, a test session was conducted to evaluate extinction retention. A second test was conducted 7, 14, 21 or 28 days later to assess spontaneous recovery (Fig. 1A).

During the extinction session, freezing levels decayed over time in all groups, indicating extinction acquisition (Repeated-measures ANOVA, F_(5,140)_ = 13.625, P = 0.001; Fig. 1B). At test 1, animals exhibited low freezing levels, indicating extinction retention (Fig. 1C). To assess spontaneous recovery, performance at test 1 and test 2 were compared with a repeated measures ANOVA, which revealed a significant group x session interaction (Repeated-measures ANOVA, F_(3,27)_ = 8.085, P = 0.0005). Tukey’s *post-hoc* showed that there was significant recovery of fear responses only in groups where test 2 was conducted either 21 (P = 0.013) or 28 days (P = 0.0002) after test 1, but not earlier (7 days: P = 0.999; 14 days: P = 0.969; Fig. 1C).

The results illustrate the well described^15^ time-dependent spontaneous recovery of fear memory following extinction. In our protocol, extinction memory suppresses fear responses for at least 14 days. After 14 days, spontaneous recovery of fear can take place. Hence, although initially dominant, extinction memory decays with the passage of time allowing for the resurgence of fear expression.

### Experiment 2: Periodical reactivations of extinction trace delays its time-dependent decay (spontaneous recovery)

In experiment 1 we found that initially extinction memory is dominant over the aversive memory trace, preventing its expression. This effect fades at later time-points when extinction is no longer able to suppress fear expression. It has been reported that reactivation sessions can lead to memory strengthening^30, 36, 39^. Since the extinction trace decays over time, we predicted that its reactivation would possibly result in its strengthening, thus enhancing its persistence and ability to suppress fear at remote time-points.

**Figure 2:**
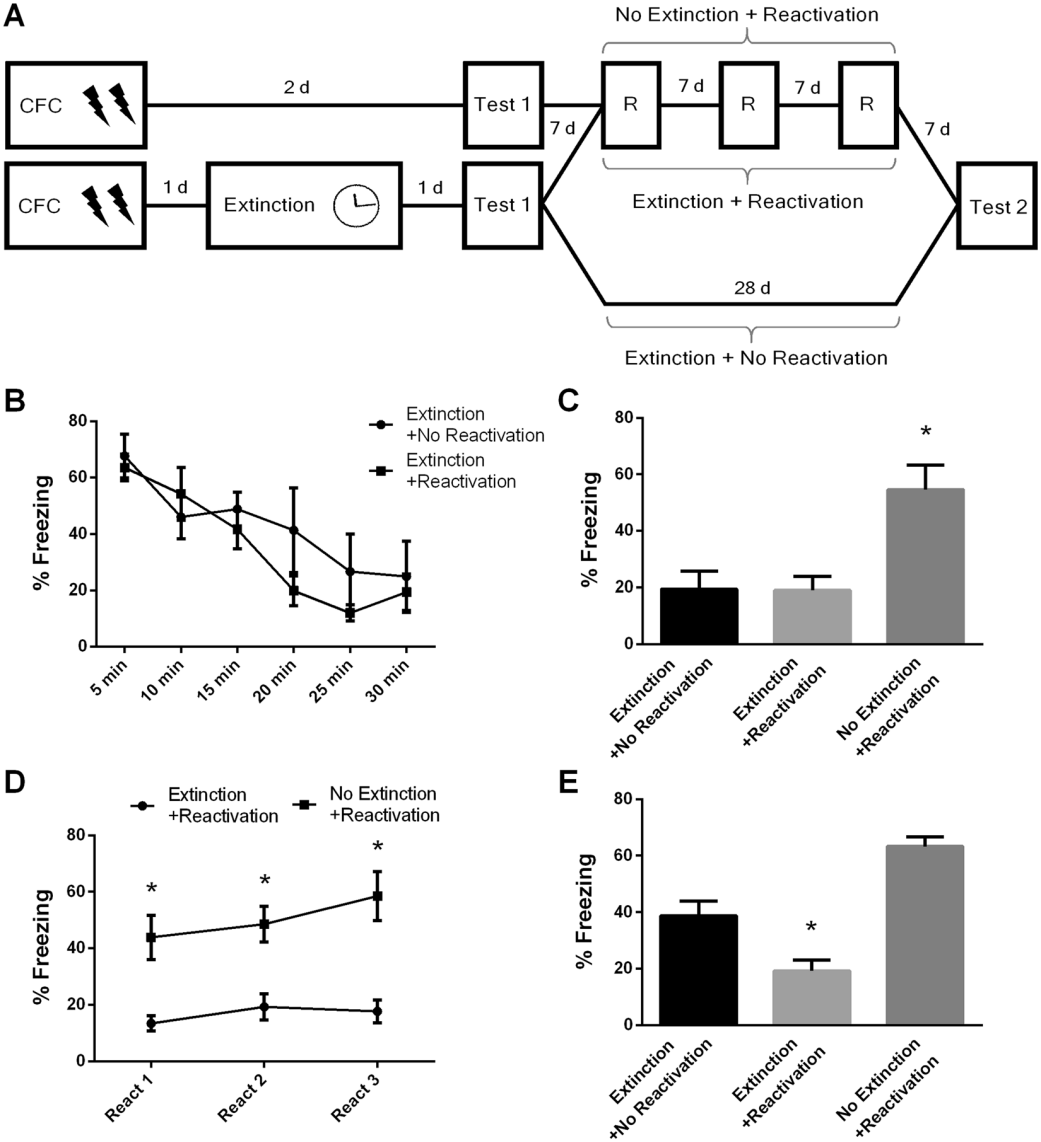
Periodical reactivations prevent spontaneous recovery of extinction memory. (**A**) Schematic representation of the experimental procedures. Fear conditioned rats were submitted to 30-min extinction session or remained in their homecages (No extinction + Reactivation group: N = 7). A test was conducted in the next day and a retest 28 days later. In the interval between tests, animals were reactivated by 3-min on days 7, 14 and 21 after test 1 or remained in their homecages (Extinction + No reactivation group: N = 10; Extinction + Reactivation group: N = 14). (**B**) Freezing levels during extinction sessions. (**C**) Freezing levels during test. (**D**) Freezing levels during reactivations. (**E**) Freezing levels during retest. (*) Significant differences between groups (P < 0.05, Two-Way or Repeated-Measures ANOVA followed by Tukey’s *post-hoc* test).

Accordingly, fear-conditioned rats underwent extinction training, were tested 1 day later and retested 28 days later for spontaneous recovery. In the interval between test and retest, a group of animals underwent 3-min reactivation sessions on days 7, 14 and 21 days after test 1 (Reactivation group) or remained in their homecages (Control group; Fig. 2A). An additional group was submitted to reactivation sessions but no extinction training (No extinction + reactivation group). The 7 day interval between reactivations was chosen because at this time-point, extinction is still robustly expressed (Experiment 1).

During extinction training, animals effectively displayed a time-dependent decrease of fear responses (F_(5,110)_ = 30.516, *P* = 0.001; Fig. 2B). On test 1, there was significant difference between groups (F_(2,28)_ = 8.11, *P* = 0.002; Fig. 2C). Tukey´s *post-hoc* showed that Control and Reactivation groups displayed similar freezing levels (P = 0.915) and both showed lower freezing than the No extinction + reactivation group (P = 0.007 and P = 0.001, respectively). During reactivation sessions, Repeated-measures ANOVA showed a significant difference between Reactivation and No Extinction + reactivation groups (F_(1,19)_ = 46.63, *P* = 0.0001) and no effect of session (F_(2,38)_ = 2.28, *P* = 0.116) nor group x session interaction (F_(2,38)_ = 1.00, *P* = 0.376; Fig. 2D). Comparison between test 1 and test 2 revealed significant group x session interaction (Repeated-measures ANOVA, F_(2,28)_ = 3.89, *P* = 0.03). Tukey´s *post-hoc* showed that fear in the Reactivation group and in the No extinction + reactivation group did not change from test 1 to test 2 (P = 0.844), but there was significant fear recovery in the control group (P = 0.02). Importantly, freezing of Reactivation group was lower than all others on test 2 (Reactivation x Control: P = 0.007; Reactivation x No extinction + reactivation: P = 0.0001; Fig. 2E). This shows that reactivation strengthened extinction memory and thus prevented fear recovery at a remote time-point. Notably, in this protocol, reactivation sessions per se had no effect on fear levels when no extinction learning took place.

### Experiment 3 - Reactivation-induced strengthening of extinction depends on L-VGCCs

**Figure 3:**
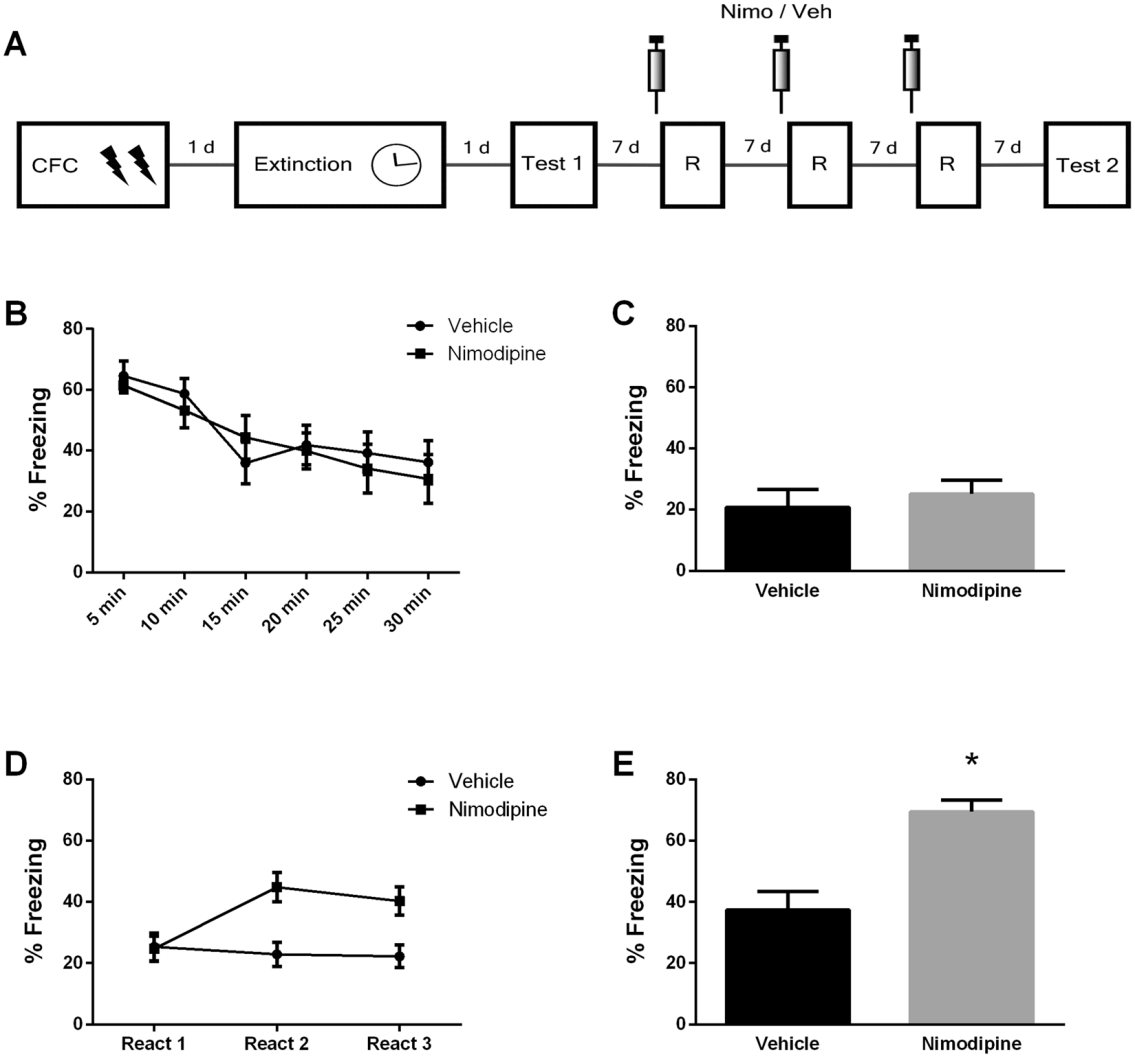
Reactivation-induced strengthening of extinction relies on L-type voltage-gated calcium channels. (**A**) Schematic representation of the experimental procedures. Fear conditioned rats were submitted to 30-min extinction session. A test was conducted in the next day and a retest 28 days later. In the interval between tests, animals were reactivated by 3-min on days 7, 14 and 21 after test 1. Nimodipine (N = 15) or its vehicle (N = 12) were s.c. infused 30 min prior each reactivation (**B**) Freezing levels during extinction session. (**C**) Freezing levels during test. (**D**) Freezing levels during reactivations. (**E**) Freezing levels during retest. (*) Significant differences between groups (P < 0.05, Two-Way or Repeated-Measures ANOVA followed by Tukey’s *post-hoc* test).

In order to induce reconsolidation, memory must be reactivated and enter into a labile state. Previous works have shown that the activation of L-type voltage-gated calcium channels (L-VGCC) during reactivation is required for destabilization, and their blockade by nimodipine prevents reconsolidation from taking place^51^. To assess the role of L-VGCC in the reactivation-induced strengthening of extinction, we repeated the experimental design of the last experiment and administered nimodipine before reactivation (Fig. 3A).

During extinction training, animals displayed a time-dependent decrease of fear responses (F_(5,125)_ = 13.55, *P* = 0.001; Fig. 3B). On test 1, animals from the Vehicle and Nimodipine groups exhibited equally low freezing levels (Student’s *t* test; *t*_(25)_ = 0.510, *P* = 0.615; Fig. 3C). During reactivation sessions, there was a significant group x session interaction (F_(2,50)_ = 7.863, *P* = 0.001; Fig. 3D) with nimodipine-treated animals exhibiting increased fear across sessions (*P* = 0.004), whereas there was no change in fear expression in vehicle-treated rats (*P* = 0.983).

At the second test, nimodipine-treated rats displayed higher freezing levels than controls (*t*_(25)_ = 5.44, *P* = 0.0001; Fig. 3E). Comparing the performance of both test sessions, a repeated-measures ANOVA found a significant group vs session interaction (F_(1,20)_ = 7.75, *P* = 0.006). Tukey’s *post-hoc* revealed that the performance of nimodipine-treated animals at test 2 was higher than the performance of all other groups and sessions (*P* < 0.001). This shows that extinction enhancement by reactivations require L-VGCC activation.

### Experiment 4: A single extinction trace reactivation opens a protein-synthesis sensitive window

**Figure 4:**
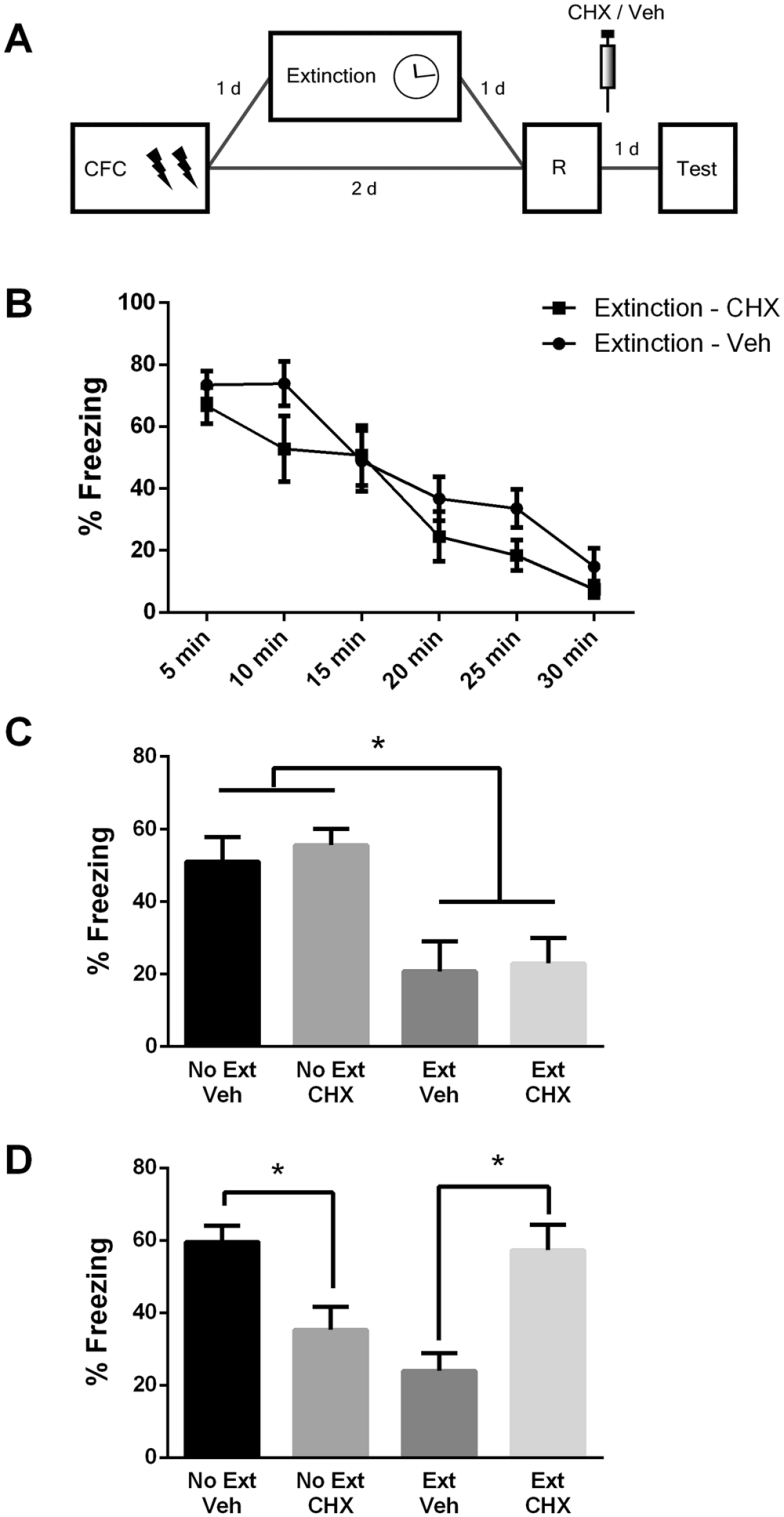
Extinction memory requires de *novo protein* synthesis after its reactivation in order to persist. (**A**) Schematic representation of the experimental procedures. Fear conditioned rats were submitted to 30-min extinction session or remained in their homecages. A reactivation session was conducted 7 days later followed by a test in the next day. Immediately after reactivation, animals received i.p. infusion of cycloheximide (Extinction group: N = 10; No-extinction group: N = 14) or its vehicle (Extinction group: N = 9; No-extinction group: N = 11). (**B**) Freezing levels during extinction session. (**C**) Freezing levels during reactivation session. (**D**) Freezing levels during test session. (*) Significant differences between groups (P < 0.05, Repeated-Measures or Two-Way ANOVA followed by Tukey’s *post-hoc* test).

Following reactivation, memory may undergo a phase that requires *de novo* protein synthesis to be reconsolidated and persist. Thus, in this labile state it is susceptible to disruption by protein synthesis inhibitors^1^. In previous work, it has been demonstrated that the extinction memory is susceptible to reconsolidation disruption by post-reactivation interference in the inhibitory avoidance paradigm^5, 6^. After employing reexposure sessions in previous experiments, we here evaluated whether these reexposures were actually a reactivation session involving the recruitment of protein synthesis. Accordingly, animals were fear conditioned and the next day a group underwent extinction training (extinction group) while others remained in their homecages (no-extinction group). On day 2 after training, all animals underwent a brief 3 min reactivation session and immediately after were injected with the protein synthesis inhibitor cycloheximide (CHX) or its vehicle. On the following day, animals were tested (Fig. 4A).

During the extinction session, a repeated-measures ANOVA revealed extinction acquisition (F_(5,75)_ = 24.08, *P* = 0.001; Fig. 4B). During reactivation, a two-way ANOVA indicated that animals that previously underwent extinction displayed lower freezing levels compared to the no-extinction group (F_(1,43)_ = 23.32, *P* = 0.001; Fig. 4C). At the test, a two-way ANOVA revealed a significant group x drug interaction (F_(1,43)_ = 22.64, *P* = 0.001). Tukey´s *post-hoc* revealed that cycloheximide-treated animals in the no-extinction group displayed lower freezing levels than vehicle-treated ones (*P* = 0.01), indicating that fear memory was impaired. In the extinction group, CHX-treated animals showed higher freezing levels than vehicle-treated ones (*P* = 0.001), indicating that extinction memory was disrupted.

Accordingly, when no extinction training was conducted, the fear trace is destabilized by reactivation and is disrupted by CHX. When the extinction and fear memories co-exist, a trace dominance effect takes place and the extinction memory is the one that suffers destabilization, requiring *de novo* protein synthesis to persist. This finding shows that the extinction trace was effectively reactivated, suggesting that reconsolidation is the mechanism mediating the extinction memory’s strengthening.

### Experiment 5 – Another extinction trace relapse process, Rapid Reacquisition, can be pharmacologically detained with a post-reactivation treatment

It has been reported that reconsolidation can be enhanced by post-reactivation administration of compounds such as HDAC inhibitors, resulting in increased performance in a post-reactivation long-term memory test^45, 52–54^. Here, we asked whether extinction could be positively modulated by post-reactivation treatment with the HDAC inhibitor sodium butyrate (NaB). We thus employed a reconditioning protocol that allows detection of relative changes in the strength of fear and extinction memory according to the ratio of fear reacquisition. For instance, usually following standard extinction procedures, reacquisition is rapid^32, 34^, but it can be slow in certain circumstances such as extensive extinction learning or weak initial conditioning^14^.

**Figure 5:**
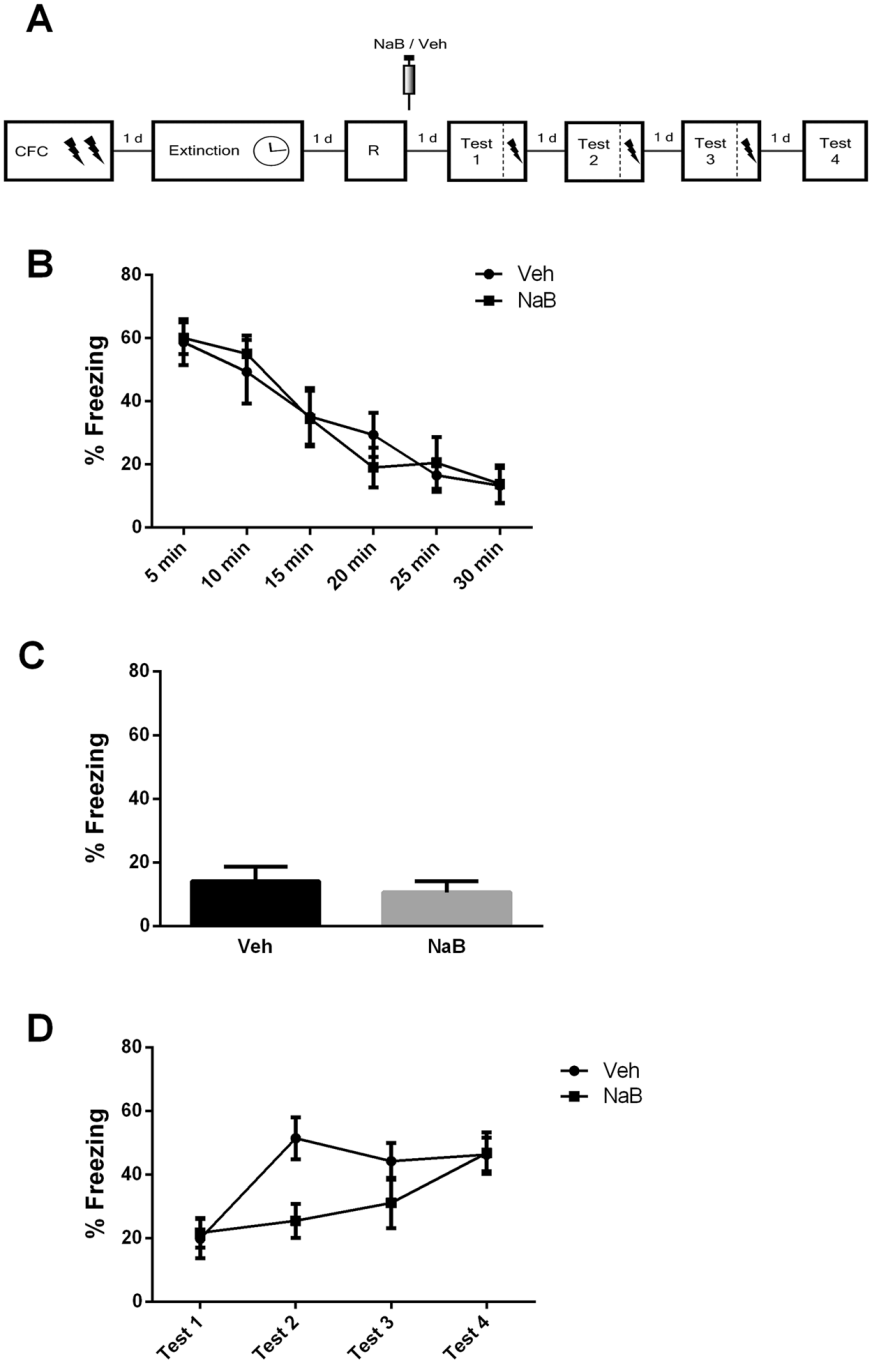
Post-reactivation infusion of NaB, a memory-enhancing drug, also strengthens extinction trace by preventing rapid reacquisition of fear. (**A**) Schematic representation of the experimental procedures. Fear conditioned rats were submitted to 30-min extinction session. A reactivation was conducted 24 h later followed by the immediate administration of sodium butyrate (NaB; N = 12) or its vehicle (N = 12). In the next 3 days, animals underwent test sessions ending with the delivery of a weak footshock plus additional 30 s of contextual exploration (rapid reacquisition protocol). One day later, a standard test was conducted. (**B**) Freezing levels during extinction session. (**C**) Freezing levels during reactivation. (**D**) Freezing levels during tests. (*) Significant differences between groups (P < 0.05, Independent-samples *t* test or Repeated-Measures ANOVA followed by Tukey’s *post-hoc* test).

Fear conditioned rats underwent extinction training and a reactivation session 24 h later. Immediately after reactivation, NaB or its vehicle was infused (i.p). In the four next days, animals underwent a mixed testing and weak reconditioning procedure to assess fear reacquisition. Each session consisted of a 4 min test followed by a weak footshock and additional 30 s period in the box (Fig. 5A). During extinction training, animals displayed a time-dependent decrease of fear responses (F_(5,110)_ = 32.89, *P* = 0.001; Fig. 5B). At reactivation, there was no difference between groups later infused with NaB or Veh (*t*_(22)_ = 0.59, *P* = 0.56; Fig. 5C). During the 4 test sessions (ending with a weak reconditioning whose effect was analyzed in the following test; Fig. 5D), repeated measures ANOVA indicated a significant drug x session interaction (F_(3,66)_ = 4.82, *P* = 0.004). Tukey’s *post-hoc* revealed that during the first test, Veh and NaB groups were equal in freezing level (*P* = 0.99). However, after one reconditioning session, Veh-treated promptly showed fear reacquisition (*P* = 0.0002) whereas NaB-treated did not (*P* = 0.99). The NaB-treated group only showed significant reacquisition after three reconditioning sessions, at test 4 (*P* = 0.002).

These results show that post-reactivation NaB treatment rendered the extinction memory resistant to rapid reacquisition. Animals from the vehicle group readily showed savings following a single weak retraining session. NaB-treated animals, on its turn, only showed savings following 3 reconditioning sessions. This demonstrates that the extinction trace can be strengthened by post-reactivation pharmacological interventions.

## Discussion

In the present study, we demonstrate that a contextual fear extinction memory can be enhanced by brief reactivation sessions. First, we showed that following extinction learning, spontaneous recovery of fear is observable 21 days after, but not 14 days or earlier (Experiment 1). Next, we found that when extinction memory was periodically reactivated, its time-dependent decay was prevented and no spontaneous recovery of fear was verified for at least 28 days (Experiment 2), an effect also shown to be mediated by L-VGCCs (Experiment 3). In order to verify if protein synthesis was being recruited by the reexposure sessions, we infused cycloheximide after reactivation and observed the occurrence of a new plasticity window, supporting the idea that reconsolidation is the process taking place (Experiment 4). Finally, employing a different protocol aimed at another relapse mechanism of extinction memory – rapid reacquisition (savings) – we verified that post-reactivation HDAC pharmacological inhibition was also able to enhance the extinction trace, as proven by the observed resistance to rapid reacquisition of fear response.

It is known that the loss of conditioned responses following extinction is not permanent^55^ since extinction does not actually rely on memory erasure. Instead, it promotes new learning which prevents the expression of the previously stored association^14^. Hence, extinction and fear memories co-exist and compete for expression. This leads to a trace-dominance effect with extinction inhibiting fear expression. However, extinction easily decays and fear memory overcomes its inhibition through several relapse mechanisms^15, 20^. The most evident of these is the return of fear by the mere passage of time, termed spontaneous recovery^15^. In addition, fear memory can become disentangled from extinction inhibition by behavioral phenomena such as rapid reacquisition, reinstatement, and renewal^14^. The decay of extinction memory over time is showed in Experiment 1. Initially, extinction suppresses conditioned fear responses. However, at remote time-points this effect vanishes, resulting in spontaneous recovery. Rapid reacquisition is observed in experiment 5. Control rats rapidly display high fear after a single weak reconditioning session. New methods designed to circumvent extinction’s poor persistence by enhancing its strength would potentially improve psychiatric treatments of fear-related disorders^48^.

Since extinction memory weakens over time and loses its ability to suppress fear^15^, preventing this decay would be beneficial. In Experiment 2, we found that the persistence of an extinction memory can be positively modulated by its simple reactivation. Accordingly, there were no signs of spontaneous recovery when animals underwent brief reactivations, even 4 weeks after extinction training. Interestingly, reactivation did not increase fear responses in animals not submitted to an extinction training as previously reported^30^, probably due to a ceiling effect. Hence, brief reactivations sessions were able to prevent extinction’s time-dependent decay in a long lasting manner.

It is often suggested that reconsolidation takes place in order to allow memory content to be updated, maintaining its adaptive relevance to better guide future behaviors. Reports of reconsolidation-driven memory updating show that it can occur through the incorporation of new information^32, 34^ or by the strengthening of existing associations^30, 35–39^. It is important to point out that even when the strengthening results from additional learning from an identical second learning trial, the destabilization-restabilization process is still required^36^. Hence, we hypothesized that the reactivation-driven strengthening effect observed in experiment 2 could be mediated by a reconsolidation process, which encompasses a reactivation-dependent destabilization phase followed by a restabilization phase that requires *de novo* protein synthesis. Previous works have shown that the L-VGCC blocker nimodipine prevents memory destabilization, thus preventing reconsolidation^51^. In experiment 3, we found that the strengthening effect of reactivation was prevented by the L-VGCC blocker nimodipine, which supports the involvement of reconsolidation in the enhancement of extinction trace. Nimodipine has also been implicated in the impairment of extinction acquisition as well as of consolidation^56, 57^. However, in our experimental protocol, nimodipine was injected several days after the extinction session took place, making its effects distinct from those obtained around the initial extinction learning. Thus, regardless which process is actually being blocked – reconsolidation or extinction - experiment 3 shows that reactivation-induced strengthening of extinction requires the activation of L-VGCC to take place.

Reconsolidation is a process in which a previously established memory is reactivated and becomes labile, requiring *de novo* protein synthesis to persist. To further address the question of whether a reactivation session induces the reconsolidation of the extinction trace, we investigated the effect of post-reactivation protein synthesis inhibition in animals that did or did not previously undergo extinction training (Experiment 4). In animals that did not undergo extinction, post-reactivation protein synthesis inhibition disrupted reconsolidation of the contextual fear memory, resulting in low freezing levels. The opposite behavioral outcome took place in the group that underwent extinction: in the test, CHX-treated animals exhibited high freezing levels, indicating that the extinction memory was disrupted. In this experiment, since extinction was disrupted by protein synthesis inhibition after a single reexposure (as confirmed in a test the following day), no extra reexposure sessions were investigated. The results of this experiment suggest that the extinction trace was hindered to a point that it was no longer able to suppress fear. In accordance with previous work^5, 7^, it indicates that reactivation did not merely promote “additional extinction learning”, but prompted the extinction trace to enter a labile state that required reconsolidation in order to persist. One must notice that either the extinction memory or the fear memory was being expressed at the time of reactivation. Accordingly, the trace that is dominantly activated by the reactivation was the one destabilized and thus impaired by the protein synthesis inhibition.

Finally, we assessed if the extinction trace could be positively modulated by post-reactivation treatment with a memory-enhancing drug. Several pharmacological agents have been shown to enhance memory consolidation and reconsolidation, including HDAC inhibitors such as sodium butyrate^45, 52, 53^. In experiment 5, we found that post-reactivation NaB treatment enhanced the extinction trace, allowing it to resist relapse by a rapid reacquisition protocol. Animals treated with NaB displayed remarkable resistance to savings after a rapid reacquisition procedure. Vehicle-treated animals showed savings after a single weak reconditioning session, whereas NaB-treated rats required 3 reconditioning sessions to show the same recovery effect. This shows that even a brief reactivation session renders an extinction trace amenable to enhancement by positive interference. Extinction enhancement by post-reactivation HDAC inhibition, as reported here, suggests that the same beneficial effect could be achieved with other memory-enhancing drugs in order to inhibit fear expression over time.

The possibility that the reactivation-dependent strengthening of extinction could have been mediated by additional extinction instead of reconsolidation must be considered. It has been reported that even short re-exposures to a context can lead to extinction when conducted at remote time-points^35^. Altho ugh the experimental protocol of that paper differs from ours in a significant number of aspects, we have included an additional experimental group to control this specific concern: in experiment 2, the “No-extinction + reactivation” group underwent the same three reactivation sessions but was not submitted to an extinction session. This group showed no fear attenuation, neither during reactivations nor the test, different from what would be expected in extinction. Additional evidence comes from experiment 4. If a brief re-exposure induced additional extinction, then the cycloheximide treatment would have disrupted only the incremental learning of that session, leaving spared what was previously stored. However, post-reactivation cycloheximide has disrupted the extinction trace, abolishing the inhibition of fear in a test conducted 24 h later. The disruption of a previously stored trace by post-reactivation protein synthesis inhibition is consistent with the reconsolidation interpretation. Thus, considering the convergence of all behavioral and pharmacological evidence obtained with the present experimental design, our results indicate that what took place during the reactivations sessions was a reconsolidation process that mediated the strengthening of extinction memory.

Fear recovery after extinction-based approaches is critical to understand in order to improve behavioral and pharmacological treatments of anxiety disorders. In fact, extinction enhancement could be considered a hallmark of psychiatric research. Here, we found that brief reactivation sessions were effective in preventing spontaneous recovery of an extinct fear memory. This effect was mediated by L-VGCCs and involves protein synthesis, strongly suggesting reconsolidation is the mechanism behind this strengthening. We also found that positive post-reactivation pharmacological modulation was able to prevent fear rapid reacquisition. Together, these findings show that extinction can benefit from reactivation-based interventions aimed to enhance its strength and persistence. It also adds to the notion that reconsolidation and extinction are not completely separate processes as current paradigms often suggest^58^. Due to the prominence of extinction-based cognitive behavioral therapies, these findings bring relevant insights to both basic and clinical research.

## Methods

### Subjects

Male Wistar rats from our breeding colony weighing 300–350 g, aged 60–70 days, were used. Animals were housed in plastic cages, four to five per cage, with water and food available *ad libitum*. All experiments were performed in accordance with national animal care legislation and guidelines (Brazilian Law 11794/2008) and approved by the University’s Ethics Committee.

### Contextual Fear Conditioning

The CFC chamber consisted of an illuminated Plexiglas box (25.0 × 25.0-cm grid of parallel 0.1-cm caliber stainless steel bars spaced 1.0 cm apart). In the conditioning session, rats were placed in the chamber for 3-min, and then received two 2-secs 0,7 mA footshocks separated by a 30-sec interval. Animals were kept in the conditioning environment for additional 30 sec before returning to their homecages.

### Memory extinction, reactivation, and test sessions

Brief or long context re-exposures were used to induce memory reactivation or extinction learning, respectively. Extinction training consisted of 30-min re-exposure to the conditioned context and always occurred 24 hours after CFC. Memory reactivation sessions consisted of 3-min re-exposure to the conditioned context. On Experiments 2 and 3, reactivations were conducted on days 7, 14 and 21 after test 1. On experiments 4 and 5 they were conducted 24 hours after extinction training.

Test sessions consisted of 4-min re-exposure to the context. On experiments 1, 2, and 3, the first test was conducted 24 hours after the extinction training and a second test was conducted 7, 14, 21 or 28 days later, in order to assess fear spontaneous recovery. On experiments 4 and 5, the test session was conducted 24 hours after reactivation in order to assess the effect of post-reactivation pharmacological manipulations.

To address rapid reacquisition (or “savings”; Experiment 5), animals underwent a 4-min test followed by a weak footshock (one 2-secs 0.4 mA). After an additional 30 seconds, they returned to their homecages. This procedure was repeated daily three times, followed by an additional test one day later. In this procedure, freezing was always scored before the footshock (that consisted of a standard test session). This allowed us to both measure performance and submit animals to a weak reconditioning session.

### Drugs

Protein synthesis inhibitor cycloheximide (CHX; Sigma) was dissolved in sterile isotonic saline with 1% dimethylsulfoxide to a concentration of 2.2 mg/mL. Cycloheximide or its vehicle was injected intra-peritonially (i.p.) immediately after the reactivation. The total volume injected was 1 mL/kg.

The L-type voltage-gated calcium channels (LVGCCs) antagonist nimodipine (Sigma) was dissolved in sterile isotonic saline with 8% dimethylsulfoxide to a concentration of 16 mg/mL. Nimodipine or its vehicle was injected subcutaneously 30 min before the reactivation sessions. The total volume injected was 1 mL/kg.

Sodium butyrate (NaB; Sigma), a histone deacetylase (HDAC) inhibitor, was dissolved in sterile isotonic saline to a concentration of 0.6 g/mL. The total volume injected was 1 mL/kg i.p., immediately after reactivation.

Drug concentrations used were chosen from previous work (CHX from^34^, Nimodipine from^34^ NaB from^54^).

### Data Analysis

Memory was measured by quantifying freezing behavior and expressed as a percentage of the total session time. Freezing was scored by an observer blind to the experimental conditions. Homoscedasticity and normality of the data distribution were confirmed with Levene’s test and Kolmogorov-Smirnov test, respectively. Extinction sessions were analyzed using Repeated-Measures ANOVA. Reactivation sessions were analyzed using Student’s t test, Two-way ANOVA, or Repeated-Measures ANOVA, followed by Tukey’s *post-hoc* test. Test sessions were analyzed using Student’s t test, Two-way ANOVA, or Repeated-Measures ANOVA, followed by Tukey’s *post-hoc* test.
